# *Candida tropicalis* distribution and drug resistance is correlated with ERG11 and UPC2 expression

**DOI:** 10.1186/s13756-021-00890-2

**Published:** 2021-03-15

**Authors:** Dan Wang, Na An, Yuwei Yang, Xianggui Yang, Yingzi Fan, Jiafu Feng

**Affiliations:** 1 Department of Laboratory Medicine, The First Affiliated Hospital of Chengdu Medical College, Chengdu, 610500 Sichuan Province China; 2 Department of Laboratory Medicine, Mianyang Central Hospital, Mianyang, 621000 Sichuan Province China

**Keywords:** *Candida tropicalis*, Drug resistance, ERG11, UPC2

## Abstract

**Background:**

*Candida tropicalis* (*C. tropicalis*) is an important opportunistic pathogenic *Candida* species that can cause nosocomial infection. In this study, we analyzed the distribution and drug susceptibility of *C. tropicalis* and the relationship between ERG11 and UPC2 expression and resistance to azole antifungal agents.

**Methods:**

*C. tropicalis* was cultured and identified by Sabouraud Agar Medium, CHROM Agar Candida and ATB tests (Bio-Mérieux, France). Total RNA was extracted from the collected strains, and the ERG11 and UPC2 mRNA expression levels were analyzed by quantitative real-time PCR.

**Results:**

In total, 2872 clinical isolates of *Candida*, including 319 strains of *C. tropicalis*, were analyzed herein; they were mainly obtained from the Departments of Respiratory Medicine and ICU. The strains were predominantly isolated from airway secretion samples, and the detection trend in four years was mainly related to the type of department and specimens. The resistance rates of *C. tropicalis* to fluconazole, itraconazole and voriconazole had been increasing year by year. The mRNA expression levels of ERG11 and UPC2 in the fluconazole-resistant group were significantly higher than they were in the susceptible group. In addition, there was a significant positive linear correlation between these two genes in the fluconazole-resistant group.

**Conclusions:**

Overexpression of the ERG11 and UPC2 genes in *C. tropicalis* could increase resistance to azole antifungal drugs. The routine testing for ERG11 and UPC2 in high-risk patients in key departments would provide a theoretical basis for the rational application of azole antifungal drugs.

## Introduction

*Candida tropicalis* (*C. tropicalis*) is widely distributed in nature and is a common colonizer of the human skin, oral cavity and digestive tract [1]. Moreover, *C. tropicalis* is also an important opportunistic pathogenic *Candida* species that can cause nosocomial infection, and its isolation rate is second to that of *Candida albicans* (*C. albicans*) [2]. Patients with high-risk factors infected with *C. tropicalis* might suffer from infections in the lung [3], urogenital tract and intra-abdomen, as well as systemic infection [4–6]. Importantly, the increasing drug-resistance of *C. tropicalis* has been reported in recent years. The global SENTRY antifungal surveillance report in 2013 showed that the resistance rate of *C. tropicalis* to fluconazole was 11.60% (in 31 countries total) [7]. Moreover, data from the China Invasive Fungal Resistance Monitoring Network (CHIF-NET) had shown that the resistance rate of *C. tropicalis* to fluconazole increased from 11.20% in 2009 to 42.70% in 2014 [8]. Therefore, the risk factors involved in *C. tropicalis* infection, the genotyping of drug-resistant strains, and the mechanism of drug resistance have caused widespread concern in recent years [9].

The resistance of *C. tropicalis* to azole antifungal drugs is mainly due to mutations and/or the overexpression of ergosterol synthase (i.e., the 14α-demethylase, 14-DM)-encoding gene ERG11, the overexpression of the MDR1 gene (from the major facilitator super (MFS)-family) [10, 11], and the overexpression of the ATP-binding cassette (ABC) transporter encoding CDR genes [12]. Among these disrupted genes, the mutation and overexpression of the 14-DM-encoding gene ERG11 have been extensively studied in relation to the drug resistance of *C. tropicalis*. The UPC2 gene encodes the zinc family transcription factor Upc2p, which exerts regulatory effects on the transcriptional level in *C. albicans* [13]. However, it is still unclear whether the UPC2 gene could regulate the expression of ERG11 in *C. tropicalis*.

In this study, the distribution and drug susceptibility of *C. tropicalis* were explored and the epidemiological characteristics of *Candida* isolates from a hospital setting were determined. Moreover, the relationship between ERG11 and UPC2 expression and resistance to fluconazole was investigated to provide a basis for disease diagnosis and treatment.

## Materials and methods

### Strain sources

The samples obtained from each clinical department of the First Affiliated Hospital of Chengdu Medical College from January 2016 to December 2019 were sent to the laboratory, and the fungi were isolated and identified based on conventional methods. For strains repeatedly obtained from the same patient at the same site, only one strain was included in the analysis.

### Fungal culture and preliminary identification

The strains were inoculated onto and cultured with Sao Paulo medium (Antu Biotechnology Co., Ltd., Zhengzhou, Henan, China). The unknown fungi that were isolated were streaked on slides and subjected to Gram staining. When spores or mycelium were observed, the strain was transfected into Chromagar color development medium (Antu Biotechnology Co., Ltd.), and then it was cultured at 35 °C for 48 h. The fungal species were initially identified according to the different colors of the colonies.

### Fungal identification and antifungal susceptibility testing

The *C. tropicalis* was identified by the yeast identification kit (colorimetric method) (Bio-Mérieux Co., Ltd., Lyon, France) and the minimum inhibitory concentration (MIC) of fluconazole, itraconazole, and voriconazole against *C. tropicalis* was determined by the yeast-like fungal susceptibility kit (microdilution method) (Bio-Mérieux Co., Ltd.), according to the Performance Standards for Antifungal Susceptibility Testing of Yeasts (CLSI M60) recommended by the Clinical and Laboratory Standards Institute (CLSI) [14]. For fluconazole, susceptibility was defined as an MIC ≤ 2 µg/ml and resistance ≥ 8 µg/ml. The quality control strain for the identification and drug-sensitivity test was *Candida parapsilosis* (ATCC22019).

### Quantitative real-time PCR

In total, 50 strains of *C. tropicalis* were randomly collected, of which, 20 strains were susceptible to fluconazole and 30 strains were resistant to fluconazole, according to the drug susceptibility test. Total RNA was extracted from exponential-phase YPD broth cultures with an RNA extraction kit (Sangon Biotechnology Co., Ltd., Shanghai, China), according to the manufacturer’s instructions. Cells were collected by centrifugation at 10,000 rpm for 1 min and then washed in DEPC-treated ddH_2_O. Then the cells were re-suspended in 600 μl Snailase Reaction Buffer and 50 μl Snailase, which was vigorously shaken. The suspension was incubated at 37 °C for 5 min, followed by the centrifugation. The supernatant was collected, and 400 μl Bufler Rlysis-Y was added. The supernatant was incubated at 65 °C for 5 min and then quickly frozen at -20 °C for 5 min. Then 200 μl Buffer YCA was added, followed by centrifugation. The supernatant was collected, and RNA was recovered by chloroform extraction, followed by ethanol precipitation, which was finally re-suspended in 50 μl DEPC-treated ddH_2_O. The cDNA template was obtained from the total RNA with the HiScript II Q RT SuperMix reverse transcription kit (Vazyme Biotechnology Co., Ltd., Nanjing, Jiangsu, China). The 2 µl total RNA, 4 μl 4 × gDNA wiper Mix and 10 μl RNase free ddH_2_O were mixed and incubated at 37 °C for 5 min. Then 4 µl 5 × HiScript II qRT SuperMix was added, which was mixed gently. Reverse transcription was performed at 50 °C for 15 min and then at 85 °C for 5 s in a thermocycler. The obtained cDNAs were diluted by 10 folds with distilled water. Quantitative real-time PCR was performed with ChamQ Universal SYBR qPCR Master Mix (Vazyme Biotechnology Co., Ltd.) on a CFX96 real-time quantitative PCR instrument (BIO-RAD, California, USA). Primers were synthesized by Sangon Biotech with the following sequences: ERG11, forward 5′-TGCCTGGTTCTTGTTGCATTT-3′ and reverse 5′-AATCGTTCAAGTCACCACCCT-3′; UPC2, forward 5′-GAGTGGAACAACAACACAACAA-3′ and reverse 5′-TAAATCCCCTAAACCTGAAAGA-3′; and ACT1, forward 5′-TTTACGCTGGTTTCTCCTTGCC-3′ and reverse 5′-GCAGCTTCCAAACCTAAATCGG-3′. The reaction conditions were as follows: 95 °C for 30 s; 95 °C for 10 s, and 60 °C for 30 s, for a total of 40 cycles. The real-time quantitative PCR quality control strain was *C. tropicalis* (ATCC750), and ACT1 was used as an internal reference [15].

### Statistical analysis

The distribution and drug sensitivity results of fungi were analyzed with WHONET 5.6 software. The distribution of *C. tropicalis* was analyzed by χ^2^ test using SPSS 24.0 software. The distribution between four years was analyzed with χ^2^ test, and the Cochran Q test was performed for the distribution of each year. The independent samples *t*-test was performed for the analysis of the ERG11 or UPC2 mRNA expression levels between the drug-susceptible and drug-resistant groups, and Spearman correlation analysis was also performed. *P* < 0.05 was considered statistically significant.

## Results

### Isolation of Candida

**Fig. 1:**
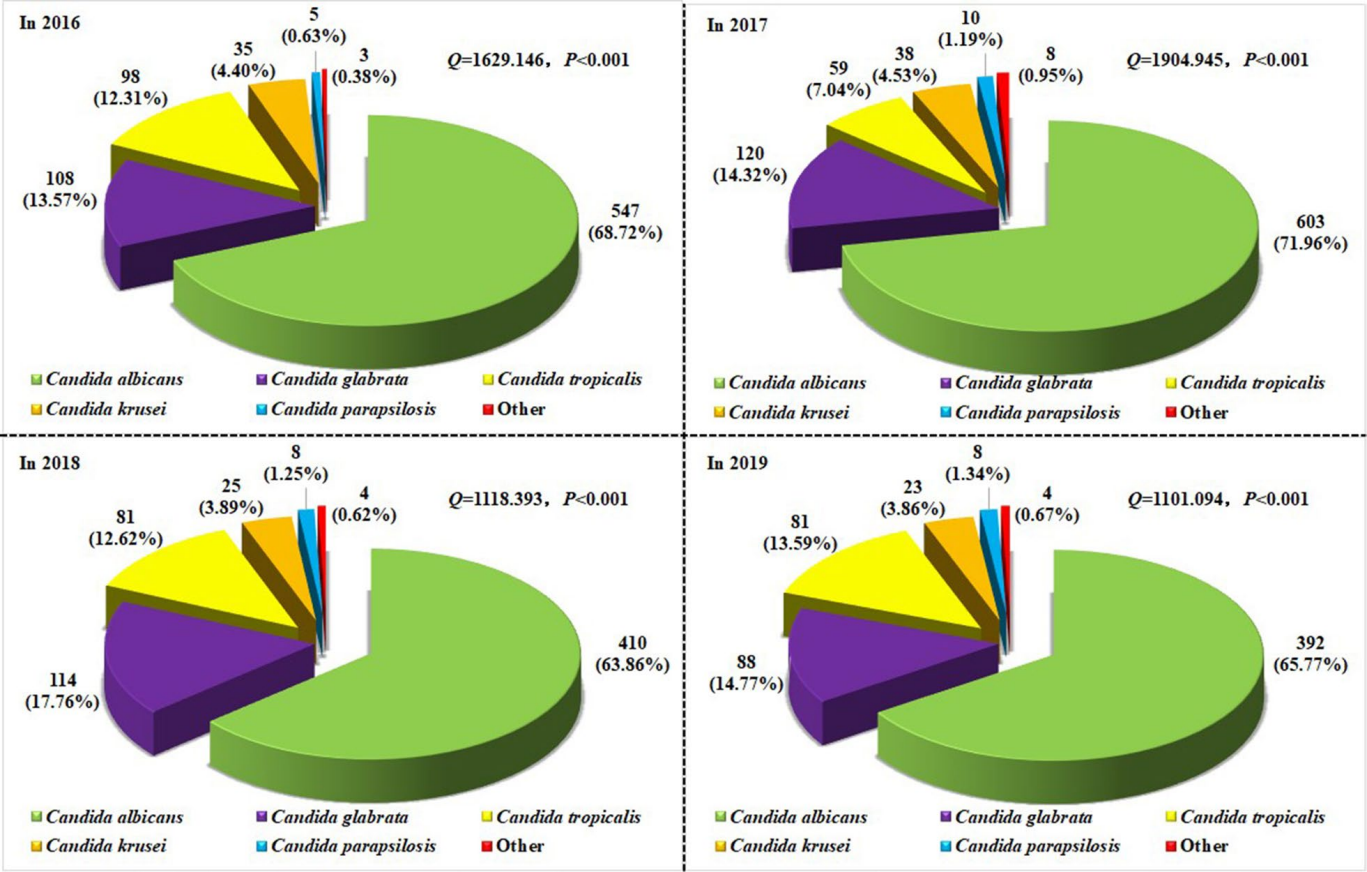
Composition of *Candida* species detected from 2016 to 2019. The composition ratios (cases and percentage) of *Candida* detected in 2016, 2017, 2018, and 2019

The distribution of various types of *Candida* strains identified in the hospital from 2016 to 2019 was analyzed. As shown in Fig. 1, a total of 2872 strains of *Candida* were identified. After the Cochran Q test, the composition ratio (i.e., the percentage of all detected *C. tropicalis* species) of various *Candida* species in each year showed statistical significance (*Q* = 1101.094–1904.945; all *P* < 0.001). For each year, *C. albicans* was the major strain, accounting for more than 60.00% of the total. *C. tropicalis* ranked third; there was no significant variation in the proportion of isolates between 2016, 2018 and 2019. Additionally, the composition ratio of each kind of *Candida* was compared between these four years. Our results showed statistical significance only for *C. albicans* (χ^2^ = 12.620; *P* = 0.006) and *C. tropicalis* (χ^2^ = 20.410; *P* < 0.001) (Additional file 1: Fig. S1).

### Sources of *C. tropicalis* samples

**Fig. 2:**
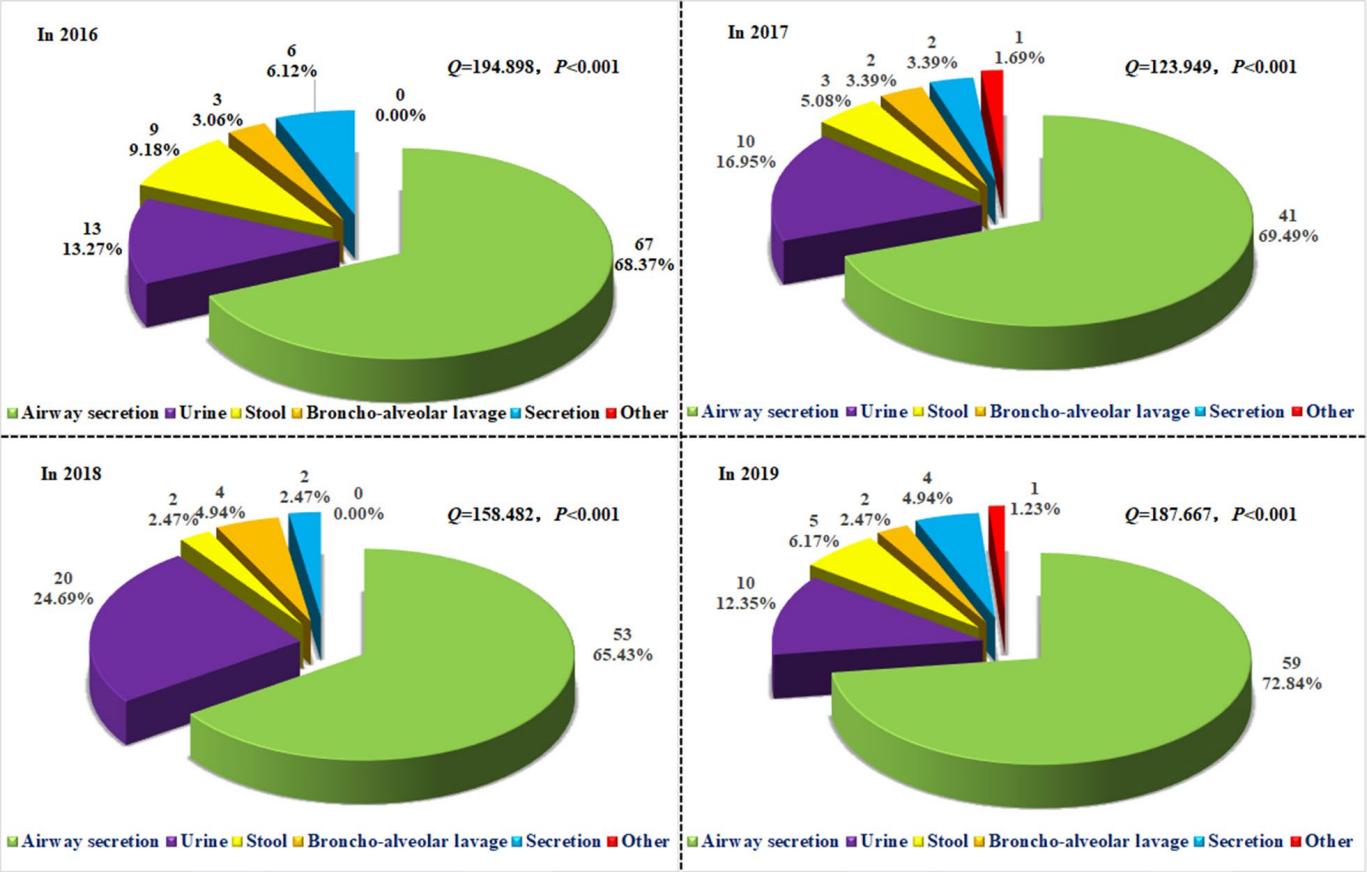
Sample composition of *C. tropicalis* from 2016 to 2019. The sample sources (cases and percentage) of *C. tropicalis* detected in 2016, 2017, 2018, and 2019

The 319 strains of *C. tropicalis* collected from 2016 to 2019 were classified according to the sample sources, and the composition ratios were also analyzed. Our results showed that significant differences were observed in the proportion of samples from which *C. tropicalis* was detected in each year (*Q* = 123.949–194.898; *P* < 0.001) (Fig. 2). Moreover, our results from the trend test showed that, except for the proportion in the stool samples (χ^2^ = 3.550; *P* = 0.060), the proportions in the airway secretions and urine samples increased over the years, but the proportions decreased for the other sample types year by year (all *P* < 0.050) (Additional file 2: Fig. S2).

### Department distribution of *C. tropicalis*

**Fig. 3:**
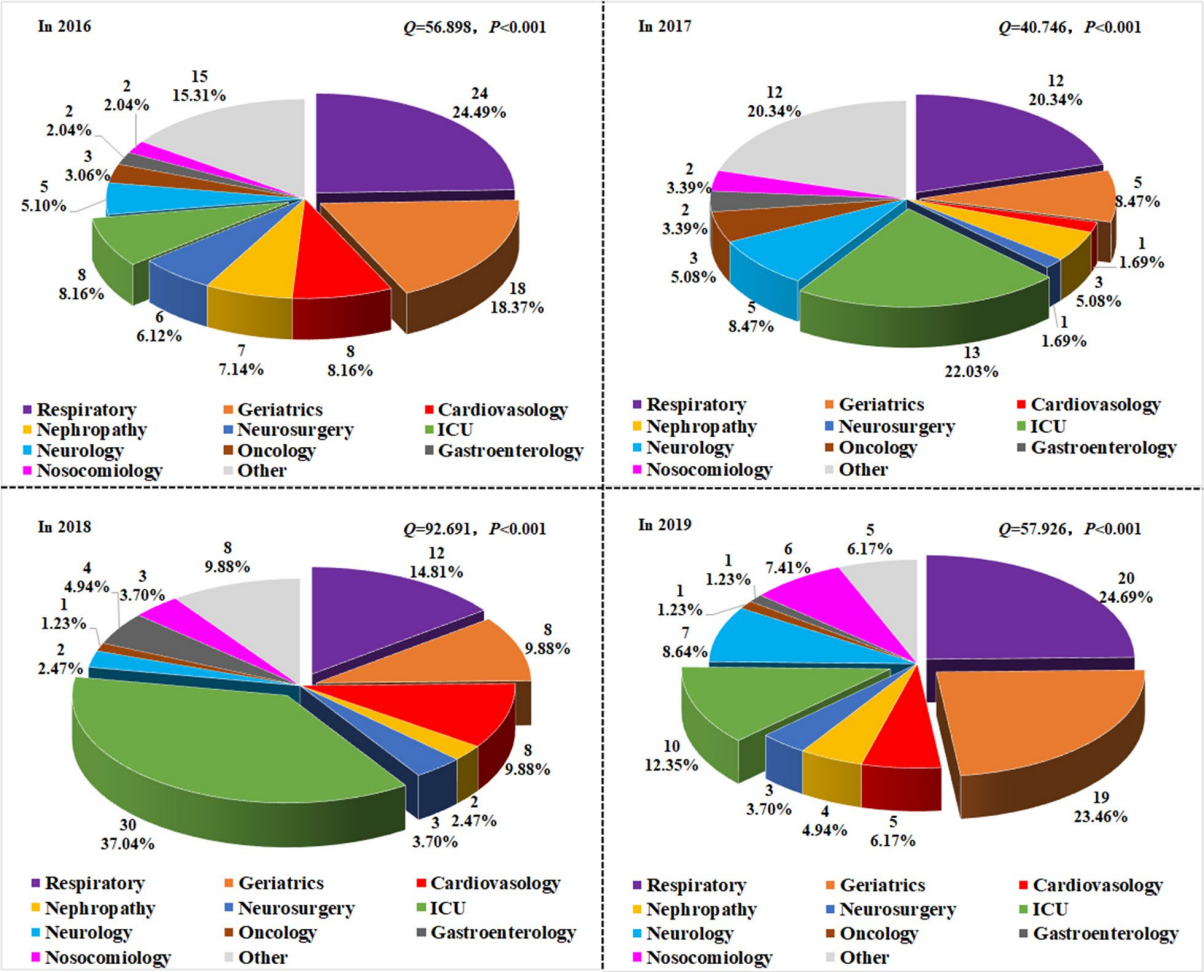
Distribution of *C. tropicalis* in different departments from 2016 to 2019. The distribution of *C. tropicalis* in different departments (cases and percentage) in 2016, 2017, 2018, and 2019

The department sources of *C. tropicalis* from 2016 to 2019 were analyzed. Our results showed that, there were significant differences in the distribution of *C. tropicalis* in these departments from 2016 to 2019 (*Q* = 40.746–92.691; all *P* < 0.001). The departments with relatively high detection rates were the Departments of Respiratory Medicine, ICU, and Geriatrics (Fig. 3). However, in comparing the *C. tropicalis* amount detected in each department in each of the four years, statistically significant differences were observed in the amount of *C. tropicalis* in the Department of Geriatrics (χ^2^ = 8.623; *P* = 0.035) and the ICU (χ^2^ = 27.148; *P* < 0.001) (Additional file 3: Fig. S3).

### Susceptibility of *C. tropicalis* to antifungal drugs

The susceptibility to azole antifungal agents including fluconazole, itraconazole and voriconazole was analyzed in the 319 samples of *C. tropicalis* collected from 2016 to 2019. Our results showed that *C. tropicalis* had high resistance rates to fluconazole, itraconazole, and voriconazole and even exhibited cross-resistance. In 2018, the resistance rate of *C. tropicalis* to fluconazole reached 39.51% (32/81) and thus this year had the most resistant *C. tropicalis*. There were no statistically significant differences in the resistance rates of *C. tropicalis* to azole antifungal drugs over these four years (χ^2^ = 1.156; *P* = 0.979). However, statistical analysis found that there were significant differences in the resistance rates of *C. tropicalis* to fluconazole (χ^2^ = 10.455; *P* = 0.015) and voriconazole (χ^2^ = 9.154; *P* = 0.027) from 2016 to 2019 (Table 1).

**Table 1:** The resistance rate of *C. tropicalis* to antifungal drugs from 2016 to 2019 (%)

Antifungal drug	2016 (*n* = 98)	2017 (*n* = 59)	2018 (*n* = 81)	2019 (*n* = 81)
Fluconazole	18.37 (18/98)	23.73 (14/59)	39.51 (32/81)	28.40 (23/81)
Itraconazole	18.37 (18/98)	28.81 (17/59)	30.86 (25/81)	23.46 (19/81)
Voriconazole	17.35 (17/98)	23.73 (14/59)	37.04 (30/81)	25.93 (21/81)

*n* is the number of strains

### Relative mRNA expression levels of ERG11 and UPC2 in *C. tropicalis*

**Fig. 4:**
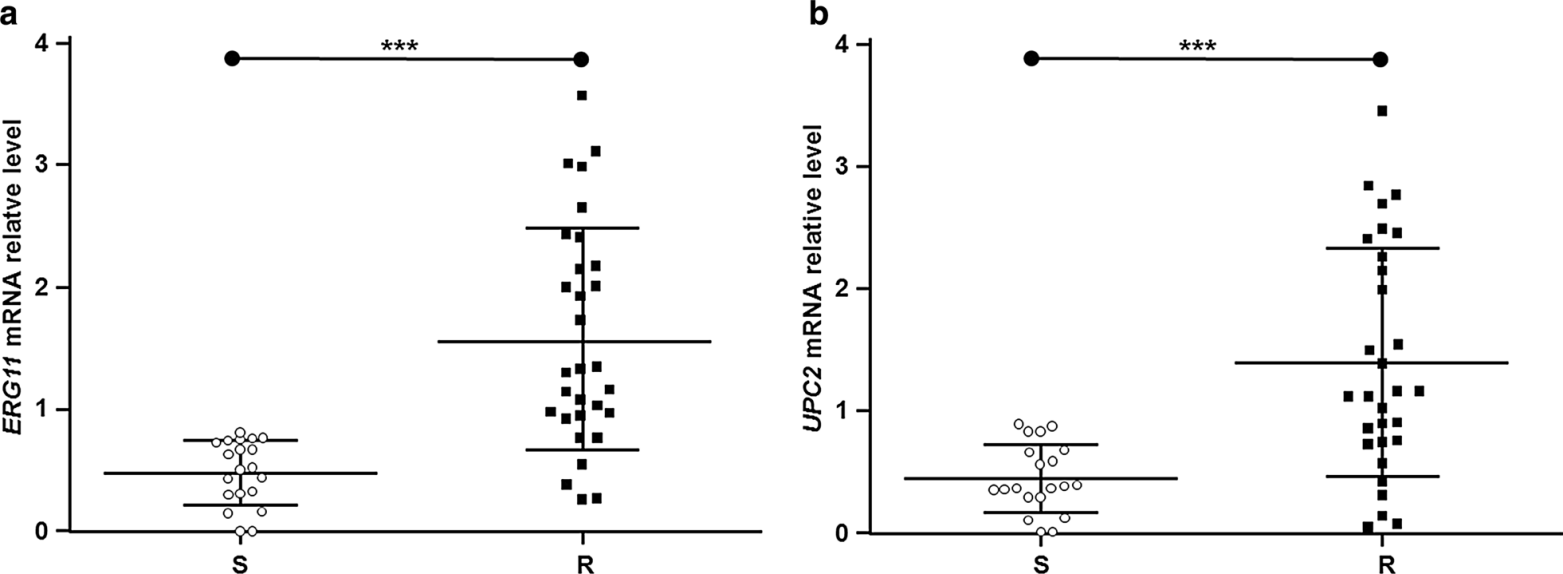
ERG11 and UPC2 expression levels in *C. tropicalis*. (**a**, **b**) Relative mRNA expression levels of ERG11 (**a**) and UPC2 (**b**) were analyzed and compared between the susceptible group (20 strains) and the resistant group (30 strains). *P* < 0.001. S, the fluconazole-susceptible group of *C. tropicalis*; and R, the fluconazole-resistant group of *C. tropicalis*

To further study the resistance-related genes of *C. tropicalis*, the mRNA expression levels of ERG11 and UPC2 were detected with quantitative real-time PCR in 20 fluconazole-susceptible and 30 fluconazole-resistant strains extracted from 319 strains. Our results showed that the relative mRNA expression levels of ERG11 and UPC2 genes in *C. tropicalis* from the fluconazole-resistant group (27/30 strains fully resistant to fluconazole, itraconazole, and voriconazole) and fluconazole- susceptible group were normally distributed. In fluconazole-resistant group, 20 strains had ERG11 overexpression and 18 strains had upregulated UPC2 expression. However, the expression level of ERG11 and UPC2 had no significant difference in the fluconazole-susceptible group. Based on the independent samples *t*-tests, the relative mRNA expression level of ERG11 in the drug-resistant group was 1.579 ± 0.896, while the relative mRNA expression of ERG11 in the susceptible group was 0.483 ± 0.259, and the difference was statistically significant (*t* = 4.511; *P* < 0.001) (Fig. 4a). On the other hand, the relative mRNA expression level of UPC2 in the resistant group was 1.400 ± 0.919, while the relative mRNA expression level of UPC2 in the susceptible group was 0.448 ± 0.272, with a statistically significant difference (*t* = 3.970; *P* < 0.001) (Fig. 4b). These results suggest that the resistance of *C. tropicalis* to fluconazole is related to the expression levels of ERG11 and UPC2.

### Correlation analysis of UPC2 and ERG11 mRNA expression in *C. tropicalis*

**Fig. 5:**
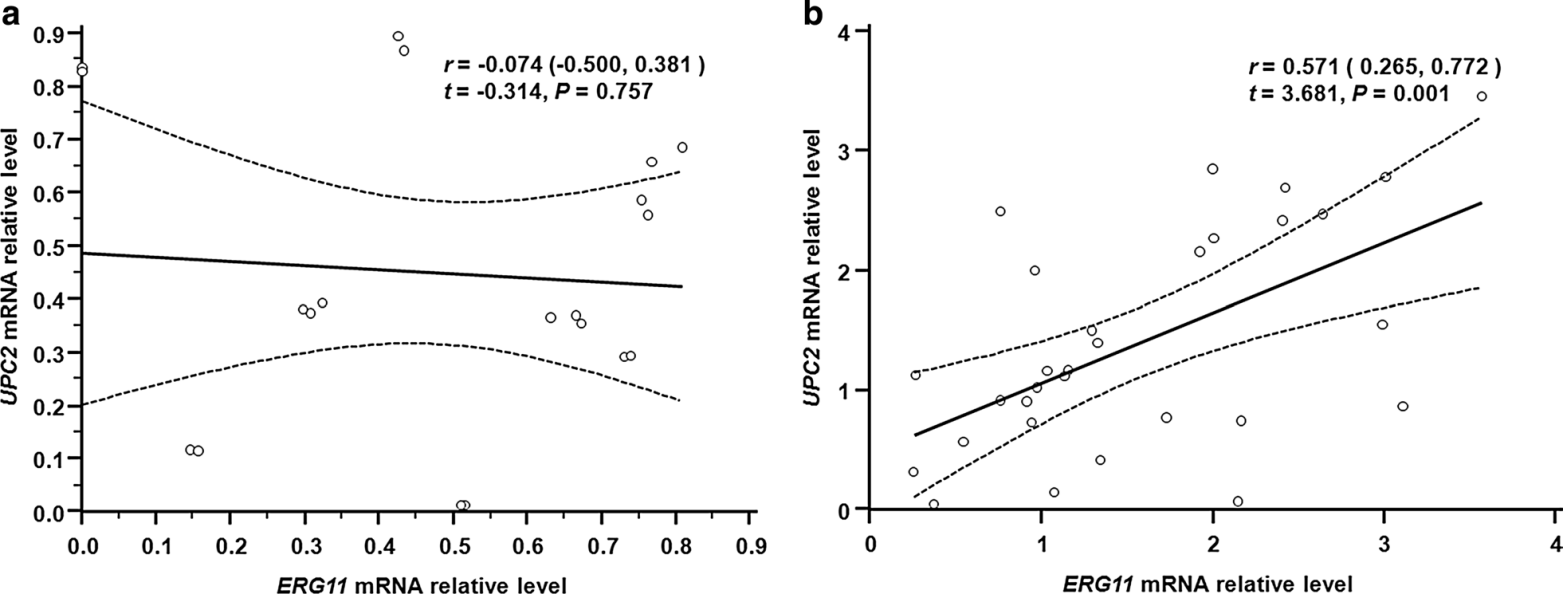
Correlation analysis between UPC2 and ERG11 expression levels. (**a**, **b**) The correlation between the UPC2 and ERG11 expression levels in the fluconazole-susceptible (**a**) and -resistant (**b**) groups of *C. tropicalis*. *P* < 0.05

The mRNA expression levels of resistance-related genes (i.e., ERG11 and UPC2) in *C. tropicalis* were detected, and the correlation between the gene expression levels was further analyzed. Our results from Spearman correlation analysis showed that there was no linear correlation between the expression levels of UPC2 and ERG11 in the susceptible group (*r* = − 0.074; *P* = 0.757) (Fig. 5a). However, the UPC2 and ERG11 expression levels were positively correlated in the drug-resistance group (*r* = 0.571; *P* = 0.001) (Fig. 5b).

## Discussion

*C. tropicalis* is a common opportunistic pathogenic fungus, and its rates of detection have been increasing over the past years. In this study, a total of 2872 strains of *Candida* were detected in samples collected from 2016 to 2019, of which *C. albicans* accounted for approximately 70.00%, while *C. tropicalis* ranked third, accounting for 12.00%. Moreover, the overall species composition of *Candida* showed statistically significant differences between the detection years. Susceptibility to antifungal agents of *C. tropicalis* in our hospital showed that the resistance rate of *C. tropicalis* to azole antifungal drugs sharply increased. In this study, based on the increasing isolation rate and drug resistance rate of *C. tropicalis*, the distribution and drug susceptibility of *C. tropicalis* were analyzed, as well as the relationship between ERG11/UPC2 gene expression and resistance to azole antifungal drugs.

Our results showed that there was no significant difference in the overall species composition from *C. tropicalis* samples tested over these four years. Moreover, the main source of *C. tropicalis* was airway secretion, which might be related to that the *C. tropicalis* is one of the upper respiratory tract-colonized fungi and can be easily isolated; further, *C. tropicalis* has been shown to form biofilms on invasive catheters (such as tracheal tubes) [16]. Therefore, *C. tropicalis* with reproductive growth is often detected in airway secretions. In view of interference factors such as *Candida* colonization, a culture of sterile tissue or fluid should be recommended in the diagnosis of bronchopulmonary candidiasis. And then the final diagnosis should be made based on clinical manifestations and other fungal-related test results of the patients. In addition, *C. tropicalis* was detected in the mid-stream urine samples, with a detection rate of 13.00%-25.00%, which had increased over the years. This phenomenon might be related to the increasing number of patients treated with invasive urinary tract operations and/or the increase in the number of mid-stream urine samples sent for examination. *C. tropicalis* has strong adhesion, penetration and destructive abilities in relation to mucous membranes, and its resistance rate to azole antifungal drugs is higher than that of *C. albicans* [17]. However, it is difficult to distinguish contamination, colonization or infection from urine culture. In the diagnosis of urogenital tract infection, clinicians should consider the patient's clinical manifestations, white blood cells/hyphae in the urine and the result of puncture culture.

In this study, our results showed that there were no significant differences in the resistance rate of *C. tropicalis* to azole antifungals between these four years, but the resistance rate had been increasing year by year (with the rate of resistance to fluconazole being as high as 39.51% [32/81]). The increased resistance rate of *C. tropicalis* to azole antifungal drugs might be related to the easy application of such drugs and the relatively mild adverse reactions [18]. In the clinic, a large number of patients with high-risk factors for invasive candidiasis were subjected to prophylaxis therapy or empirical therapy by using azole drugs to prevent the infection of *Candida* [19], however, long-term application of these drugs could easily lead to the emergence of azoles-resistant strains [20, 21].. Therefore, in order to slow the increase of azole-resistant *C. tropicalis*, the cooperation of multidisciplinary teams in hospital can make clear the diagnosis of disease, improve treatment and control antibiotic use. In addition, the diagnosis of candidiasis should be according to the high-risk factors, manifestations, etiological tests, diagnosis guidelines, etc.; the treatment of candidiasis should refer to the laboratory antifungal susceptibility test, antigen test, epidemiological characteristic, etc.; only in this way can we reasonably use azoles and avoid the prevalence of azoles-resistant *Candida* [22–24].

Moreover, in this study, the ERG11 and UPC2 genes of 50 strains of *C. tropicalis* were assessed, and our results showed that the relative expression level of the ERG11 gene in the drug-resistant group was higher than it was in the susceptible group, which is in line with the findings from Jiang et al. [25] regarding the high expression of ERG11 in fluconazole-resistant *C. tropicalis*. ERG11 overexpression could increase the amount of 14-DM in cells, which ensures ergosterol synthesis and the normal growth and reproduction of *Candida*, therefore leading to azole drug resistance [26, 27]. Moreover, Jiang et al*.* [25] cloned Y132F and S154F ERG11 mutants from *C. tropicalis* and introduced them into *Saccharomyces cerevisiae* (*S. cerevisiae*) and showed that the sensitivity of *S. cerevisiae* to azole antifungal drugs, especially fluconazole, was decreased. These results suggest that Y132F and S154F are involved in the resistance of *C. tropicalis* to fluconazole. Moreover, our results showed that the expression level of UPC2 in the resistant group was also higher than that of the susceptible group, indicating that the overexpression of UPC2 may cause *C. tropicalis* to become resistant to azole antifungal drugs, which was consistent with the findings from Jiang et al*.* [28]. In this study, the correlation analysis of the ERG11 and UPC2 mRNA expression levels in *C. tropicalis* showed that there was a linear positive correlation between the genes in the drug-resistant group. These results indicated that when UPC2 was over-expressed in the fluconazole-resistant *C. tropicalis*, ERG11 would also be over-expressed. Therefore, the expression level of ERG11 might increase with the over-expression of UPC2. It has been shown that UPC2 has transcriptional regulation in *C. albicans* [13] and the over-expression of UPC2 in the fluconazole-resistant *C. albicans* can induce the over-expression of ERG11 [29, 30]. Therefore, our results suggest that the over-expression of UPC2 in the fluconazole-resistant *C. tropicalis* may effectively promote the over-expression of ERG11, and then increase the ergosterol synthase in cell membrane and cause the resistance to azole antifungal drugs in *C. tropicalis*, especially fluconazole. However, Choi et al*.* [31] sequenced the UPC2 gene in *C. tropicalis*, and their results showed that the amino acid substitutions caused by mutations in the gene appeared not only in the resistance group overexpressing ERG11, but also in the susceptible group with no ERG11 overexpression. So far, no effective missense mutation has been detected in the UPC2 of fluconazole-resistant *C. tropicalis*, and therefore the reason for the over-expression of UPC2 needs further study. If the expression of ERG11 and UPC2 genes can be routinely detected in clinic, the resistance of *C. tropicalis* to azole antifungal drugs can be evaluated according to the gene expression levels, which might provide more valuable guidance to the treatment of *C. tropicalis* infection. Drug resistance might also be related to multiple factors, and in a few drug-resistant *C. tropicalis* without ERG11 and UPC2 overexpression, the mechanisms underlying drug resistance might be related to efflux pumps [32] and biofilm formation [33]. To fully understand the drug resistance mechanisms of *C. tropicalis*, it is necessary to comprehensively study the impacts of the mechanism on drug sensitivity. Based on these findings, further in-depth studies are still needed to investigate the transcriptional regulatory function of Upc2p in drug-resistant *C. tropicalis* and to explore how UPC2 overexpression regulates ERG11, thus leading to drug resistance to azole antifungal drugs.

## Conclusions

*C. tropicalis* has become the most common pathogen responsible for non-*C. albicans* infection, and the drug resistance rate has gradually increased. It can often cause infections in patients with low immunity, basic diseases, invasive procedures, and/or long-term and large-dosage application of broad-spectrum antibiotics. Our results showed that the fluconazole resistance of *C. tropicalis* with ERG11 overexpression may be related to the regulation of the zinc family transcription factor Upc2p. Therefore, when selecting and administering azole antifungal drugs, in addition to drug sensitivity findings, clinicians should fully understand the species distribution, the formation of drug resistance, and the overexpression of ERG11 and UPC2 genes. Routine detection of ERG11 and UPC2 for high-risk patients in the clinic would contribute to early disease diagnosis and timely treatment to delay and prevent the development of resistance to *C. tropicalis*.

## Supplementary information

**Additional file 1: Fig. S1.** Composition of various *Candida* species from 2016 to 2019. The composition ratios of each type of *Candida* detected from 2016 to 2019 were analyzed. The composition ratios of each type of *Candida* as a function of all types (%) were analyzed for each year.

**Additional file 2: Fig. S2.** Comparison of sample composition of *C. tropicalis* from 2016 to 2019. The sample compositions of *C. tropicalis* detected from 2016 to 2019 were analyzed. The sample ratios of *C. tropicalis* as a function of all samples (%) were analyzed for each year.

**Additional file 3: Fig. S3.** Comparison of distribution of *C. tropicalis* in different departments from 2016 to 2019. The distribution of *C. tropicalis* in different departments from 2016 to 2019 was analyzed. The composition ratios of *C. tropicalis* as a function of all samples (%) in each department were analyzed for each year.

## Data Availability

The data analyzed in this study are included in the manuscript.
