# Supplementary figures and images for: Candida tropicalis distribution and drug resistance is correlated with ERG11 and UPC2 expression
Source: Antimicrob Resist Infect Control. 2021 Mar 15;10:54. doi: 10.1186/s13756-021-00890-2 (PMC7958445; doi:10.1186/s13756-021-00890-2)

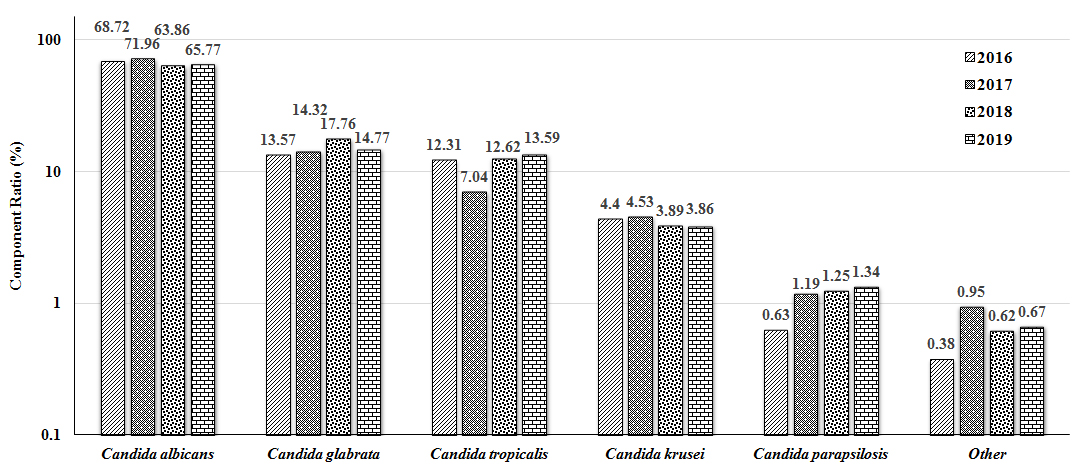

Supplement: Supplementary file 1 — Additional file 1: Fig. S1. Composition of various Candida species from 2016 to 2019. The composition ratios of each type of Candida detected from 2016 to 2019 were analyzed. The composition ratios of each type of Candida as a function of all types (%) were analyzed for each year. [file 13756_2021_890_MOESM1_ESM.jpeg]

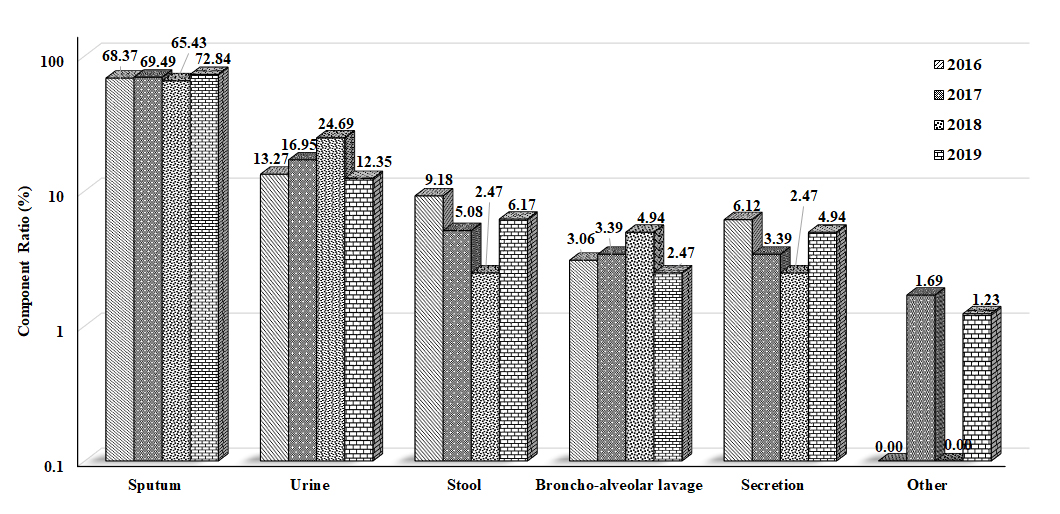

Supplement: Supplementary file 2 — Additional file 2: Fig. S2. Comparison of sample composition of C. tropicalis from 2016 to 2019. The sample compositions of C. tropicalis detected from 2016 to 2019 were analyzed. The sample ratios of C. tropicalis as a function of all samples (%) were analyzed for each year. [file 13756_2021_890_MOESM2_ESM.jpeg]

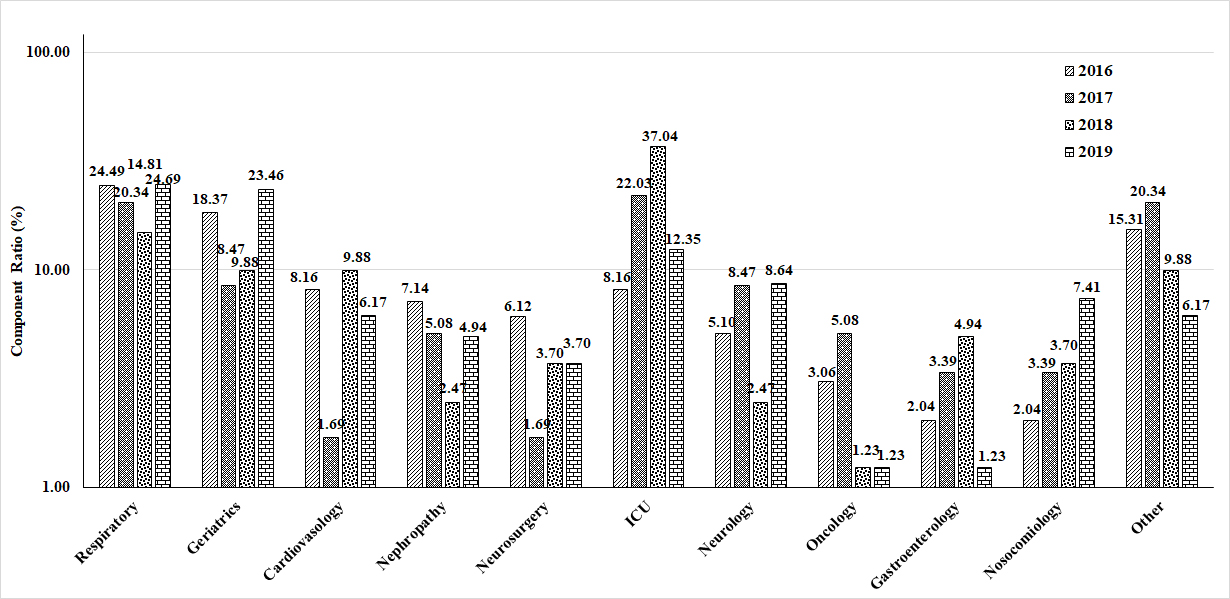

Supplement: Supplementary file 3 — Additional file 3: Fig. S3. Comparison of distribution of C. tropicalis in different departments from 2016 to 2019. The distribution of C. tropicalis in different departments from 2016 to 2019 was analyzed. The composition ratios of C. tropicalis as a function of all samples (%) in each department were analyzed for each year. [file 13756_2021_890_MOESM3_ESM.jpeg]
